# The Therapeutic Role of Monocytes in Alzheimer's Disease: Effects on Vascular Amyloid‐Beta Transport

**DOI:** 10.1002/alz70859_101318

**Published:** 2025-12-25

**Authors:** Ayanna Arneaud, Serge Rivest, Nataly Laflamme

**Affiliations:** ^1^ Uniersité Laval, Québec, QC Canada; ^2^ Université Laval, Québec, QC Canada

## Abstract

**Background:**

Alzheimer's disease (AD) is a neurodegenerative disease with no cure, and amyloid‐beta (AB) accumulation is one of its key pathological hallmarks. Cerebral Amyloid Angiopathy (CAA), characterized by AB deposition in cerebral vasculature, is increasingly recognized as a significant contributor to Alzheimer's Disease development. Monocytes, which are key innate immune cells, play a crucial role in AB clearance. However, their therapeutic potential remains underexplored in the context of CAA.

We hypothesize that the activation of the Nucleotide‐binding oligomerization domain 2 (NOD2) receptor by Muramyl Dipeptide (MDP) to convert classical monocytes into non‐classical, patrolling monocytes, will enhance AB clearance in APP/PS1 mice. Additionally, this favourable effect will be further amplified in combination with a glucocorticoid such as dexamethasone (Dex) due to its ability to upregulate key efflux transporters ABCB1 and LRP‐1 while providing anti‐inflammatory properties concurrently. In order to address this hypothesis, we'll start by investigating the implications of this NOD2 receptor and glucocorticoids on the modulation of monocytes and memory. We will also investigate the mechanisms of AB clearance.

**Method:**

We used wild‐type, NOD2 knockout (KO), and APP/PS1 transgenic mouse models to assess the effects of MDP and Dex on monocyte modulation, transporter expression, and cognitive function. Flow cytometry was used to analyze monocyte populations, while transporter expression was evaluated by western blot. Memory was assessed using the Novel Object Recognition Task.

**Result:**

MDP induced a switch from classical (LyChigh) to patrolling monocytes (Ly6Clow), suggesting enhanced AB clearance, an effect that was absent in our NOD2 KO mice. Dex treatments significantly increased ABCB1 and LRP‐1 expression in APP/PS1 mice, suggesting improved AB efflux across the blood‐brain barrier (BBB). Notably, the combination of MDP and Dex resulted in a synergistic effect, significantly enhancing transporter expression and delaying memory decline in APP/PS1 mice.

**Conclusion:**

These findings highlight NOD2 as a promising therapeutic target for modulating innate immune responses to enhance AB clearance. Additionally, the combination of NOD2 activation and glucocorticoids offers a novel dual approach to AD and CAA treatments by promoting both immune and BBB‐mediated clearance of AB while reducing inflammation.

